# Preparation of Uniform-Sized and Dual Stimuli-Responsive Microspheres of Poly(*N*-Isopropylacrylamide)/Poly(Acrylic acid) with Semi-IPN Structure by One-Step Method

**DOI:** 10.3390/polym8030090

**Published:** 2016-03-17

**Authors:** En-Ping Lai, Yu-Xia Wang, Yi Wei, Guang Li

**Affiliations:** 1 State Key Laboratory for Modification of Chemical Fibers and Polymer Materials, College of Materials Science and Engineering, Donghua University, Shanghai 201620, China; nemodhu@163.com (E.-P.L.); lig@dhu.edu.cn (G.L.); 2 National Key Laboratory of Biochemical Engineering, Institute of Process Engineering, Chinese Academy of Sciences, Beijing 100190, China; ywei@ipe.ac.cn

**Keywords:** poly(*N*-isopropylacrylamide) (PNIPAM), semi-interpenetrating polymer network (semi-IPN), stimuli-responsive, uniform microspheres, premix membrane emulsification

## Abstract

A novel strategy was developed to synthesize uniform semi-interpenetrating polymer network (semi-IPN) microspheres by premix membrane emulsification combined with one-step polymerization. Synthesized poly(acrylic acid) (PAAc) polymer chains were added prior to the inner water phase, which contained *N*-isopropylacrylamide (NIPAM) monomer, *N*,*N*′-methylene bisacrylamide (MBA) cross-linker, and ammonium persulfate (APS) initiator. The mixtures were pressed through a microporous membrane to form a uniform water-in-oil emulsion. By crosslinking the NIPAM in a PAAc-containing solution, microspheres with temperature- and pH-responsive properties were fabricated. The semi-IPN structure and morphology of the microspheres were confirmed by Fourier transform infrared spectroscopy (FTIR), scanning electron microscopy (SEM), and transmission electron microscopy (TEM). The average diameter of the obtained microspheres was approximately 6.5 μm, with Span values of less than 1. Stimuli-responsive behaviors of the microspheres were studied by the cloud-point method. The results demonstrated that semi-IPN microspheres could respond independently to both pH and temperature changes. After storing in a PBS solution (pH 7.0) at 4 °C for 6 months, the semi-IPN microspheres remained stable without a change in morphology or particle size. This study demonstrated a promising method for controlling the synthesis of semi-IPN structure microspheres with a uniform size and multiple functionalities.

## 1. Introduction

Stimuli-responsive polymers have attracted extensive interest due to their special response behavior to external environment changes. Poly(*N*-isopropylacrylamide) (PNIPAM) is one of the prominent thermally reversible polymers that can change its characteristics around the lowest critical solution temperature (LCST, approximately 32 °C in water) [1,2]. PNIPAM microspheres possess a small size and rapid response time and thus have potential use in many high-tech fields including medical diagnostics, controlled drug delivery and enzyme immobilization [3,4,5,6,7,8]. To broaden its applicability, dual stimuli-responsive PNIPAM-based microspheres have been developed. Among these, the microspheres that respond to both pH and temperature simultaneously are expected to be widely used.

The pH/temperature dual stimuli-responsive behavior of PNIPAM-based microspheres can be obtained in different ways, including the copolymerization with hydrophilic/hydrophobic monomers and the formation of interpenetrating polymer networks (IPNs) [9,10,11,12]. However, a disadvantage of the copolymerization method is that the property of each component will interfere with the other [13]. Compared with copolymerization, the formation of IPNs is an effective strategy to overcome this weakness as the individual components show little interference with each other [14]. IPNs are well known as a combination of two polymers in network form with the polymer components entangled physically [15]. Full-IPNs and semi-IPNs are the two main types of IPNs, and the full-IPNs are characterized by the presence of both polymers in the cross-linked state. If one of the components in IPNs is linear and the other is crosslinked, semi-IPNs can be formed. In both semi-IPN and IPN from two-component systems, each phase evolved by the phase separation is forced to form a compatible metastable structure [16].

Generally, PNIPAM-based IPN microgels are prepared by a two-step seed-swelling polymerization. In 2004, Hu and Xia first prepared PNIPAM–PAAc IPN nanogels (approximately 200 nm) using the two-step method [17,18]. Initially, the PNIPAM cross-linked nanogels were synthesized as the seeds for the next polymerization. The PNIPAM nanogels were then immersed in a solution containing AAc monomer and cross-linker, and the IPN structure was formed by *in situ* polymerization of AAc inside the PNIPAM network. The obtained IPN nanogels were found to undergo the volume phase transition at 34 °C, which was similar to the temperature for the PNIPAM nanogels. Subsequently, several studies have been reported on the preparation of PNIPAM-based microgels with IPN or semi-IPN structures. Ma *et al.* utilized a similar two-step method to prepare semi-IPN PNIPAM–PAAc nanocomposite microgels [19]. First, the PNIPAM microgel was prepared via surfactant-free emulsion polymerization using clay as a cross-linker, and then the AAc monomer was polymerized to form PAAc chains within the PNIPAM microgels. The obtained semi-IPN microgels with diameters ranging from 360 to 400 nm could respond independently to both pH and temperature changes. Temperature and pH dual stimuli-responsive hollow nanogels with an IPN structure based on a PAAc network and a PNIPAM network were fabricated by a two-step sequential colloidal template polymerization and the subsequent removal of the cavity templates [20,21]. These microcapsules were approximately 500 nm at room temperature, and possessed pH and temperature stimuli-responsive properties with little mutual interference. Ahmad *et al.* prepared IPN hydrogel microspheres based on PNIPAM and poly(methacrylic acid) (PMAA) by a sequential polymerization method [22]. The prepared microspheres with a diameter of approximately 400 nm exhibited both temperature- and pH-sensitive volume phase transitions.

In summary, the resultant particles mentioned above exhibited improved temperature and pH responsiveness. However, the two-step method was complicated in terms of the production of the IPN or semi-IPN particles. The polymerization time needed to be controlled carefully, otherwise the structures of particles would change from IPN to core–shell structures [17,23]. Additionally, the sizes of the obtained spheres were nano- or sub-micron, which will limit their further applications. For instance, particles are often filled into columns for separation in protein analysis processes, and extremely small spheres will generate high back pressure [24]. Furthermore, it is inconvenient to identify the structure of nano-sized particles in aqueous solution using optical microscopy. Because the properties and behaviors of the microgels are strongly dependent on its water content, a direct inspection of microgels in aqueous solution is very important [25]. Given the above considerations, it would be beneficial to explore an easier strategy for fabricating uniform dual stimuli-responsive micro-sized microspheres with an IPN or semi-IPN structure. Membrane emulsification is a technique that has proven especially useful in preparing uniform-sized particles [26]. According to the preparation mechanisms, this technique is divided into two types: direct membrane emulsification and premix membrane emulsification. Compared with the former, it is more suitable to prepare small particles for the systems with high viscosity in the premix membrane emulsification process. Moreover, the technique characterized by a high trans-membrane flux can obtain uniform droplets efficiently, which would be beneficial for industry production in large scale. Recently, our research group has prepared uniform particles with various structures using this technology [27,28,29]. Therefore, it is expected that dual stimuli-responsive PNIPAM-based microspheres with semi-IPN structure can be prepared by this novel process.

In this study, we proposed combining the premix membrane emulsification process with the subsequent one-step suspension polymerization method to fabricate dual stimuli-responsive microspheres with a semi-IPN concept. Thus, by carrying out polymerization of NIPAM with cross-linker in PAAc aqueous droplets, the two-component network could be formed directly. This work was focused on the preparation and characterization of semi-IPN structure microspheres, as well as its temperature- and pH-induced volume phase transition behaviors. Furthermore, the stability of microspheres stored in PBS solution was investigated using confocal laser scanning microscopy (CLSM) and a laser particle size analyzer at different times.

## 2. Experimental Section

### 2.1. Materials

*N*-isopropylacrylamide (NIPAM) was purchased from Tokyo Chemical Industrial Co., Ltd. (Tokyo, Japan) and was recrystallized from *n*-hexane at 40 °C before using. Acrylic acid (AAc) and Span 80 were bought from Sinopharm Chemical Regent Beijing Co., Ltd. (Beijing, China), and ammonium persulfate (APS) was supplied by Shantou Xilong Chemical Factory Guangdong, China. *N*,*N*,*N*′,*N*′-tetramethylethylenediamine (TEMED), *N*,*N*′-methylenebis (acrylamide) (MBA) and Rhodamine 123 (Rh 123) were purchased from Sigma-Aldrich Co., St. Louis, MO, USA. All these reagents of analytical grade were used as received. The micro-porous membrane and membrane emulsification equipment (FM-500M) were kindly provided by Senhui Microsphere Tech (Suzhou, China) Co., Ltd. Deionized water used in the synthesis and characterization was obtained through the RiOs-water system (Millipore Corp., Billerica, MA, USA) to remove impurities.

### 2.2. Synthesis of PAAc

Six milliliters of acrylic acid, 0.45 g of APS, and 0.5 g of isopropyl alcohol were added into 15 mL of water, after which the mixture was heated to 80 °C. Polymerization was carried out for 2 h in a 100-mL three-neck flask attached to a reflux condenser. The PAAc solution was washed with petroleum ether to remove impurities and then dried at room temperature in a vacuum. Gel permeation chromatography (GPC, Waters Corp., Milford, MA, USA) was used to measure the molecular weight of the polymer using polystyrene as a standard, with 5800 as the number-average molecular weight (*M*_n_) of the PAAc polymer and 1.08 as the *M*_w_/*M*_n_ value.

### 2.3. Preparation of PNIPAM and Semi-IPN Microspheres

**Figure 1:**
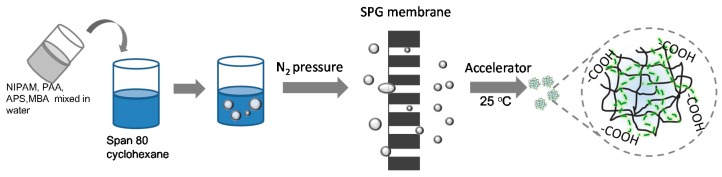
Process for the preparation of PNIPAM/PAAc semi-IPN microspheres.

The microspheres were synthesized using suspension polymerization (Figure 1), and the recipes of the water phase are shown in Table 1. Generally, different ratios of NIPAM, PAAc, MBA, and APS were dissolved together in 9.5 mL of deionized water. The pH value of the water phase was adjusted to 4.0 using an HCl solution, and the carboxyl groups of PAAc were not ionized as its pKa was 4.7 [30]. In this pH condition, a hydrogen bond could exist between the carboxyl groups of PAAc and the amide groups of NIPAM. Cyclohexane (100 mL) and 2.5 g of Span 80 were mixed together in a beaker. The water phase was then dispersed into the oil phase at 140 rpm for 30 min. The coarse emulsion was pressed through a micro-porous membrane (the pore size of the membrane was 5.2 μm) under a certain nitrogen pressure, and this preliminarily emulsified emulsion was passed through the same membrane in the next pass. The final water-in-oil (W/O) emulsion was obtained after repeating the above process 1–5 times. The obtained emulsion was bubbled with nitrogen gas to remove oxygen before polymerization. Afterwards, TEMED dissolved in 2 mL of cyclohexane was introduced to accelerate the reaction. The system was kept at 20 °C under a nitrogen atmosphere for 4 h.

After the polymerization, the dispersions were washed with acetone and deionized water three times to ensure the complete removal of unreacted chemicals. The obtained microspheres were dispersed in deionized water for further analysis. A series of PNIPAM–PAAc semi-IPN microspheres (semi-IPN1, semi-IPN2, semi-IPN3) were synthesized by changing the initiator APS amount while keeping a constant molar ratio of initiator APS to accelerator TEMED.

### 2.4. Characterizations of Microspheres

The shape and surface morphology of the microspheres were observed with a scanning electron microscope (SEM, JEOL, JSM-6700F, Tokyo, Japan). Before the measurement, the microspheres were dried by CO_2_ supercritical drying with K850 Critical Points Driers (Quorum/Emitech, Ashford, UK) [11]. The resulting samples were coated with gold using a fine coater (JEOL JFC-1600, Tokyo, Japan) on a copper platform.

The morphology of the dried microspheres was examined by transmission electron microscopy (TEM, JEOL, JME-2100, Tokyo, Japan). Before TEM observation, the samples were stained by mixing 1 mL of the microsphere dispersion and 100 μL of a 0.75 mmol·L^−1^ uranyl acetate solution. Subsequently, 15 μL of the mixture was placed on a copper grid (coated with a carbon membrane) and then dried overnight at room temperature.

The particle size and size distribution was measured by laser diffraction using a Mastersizer 2000 (Malvern Instrument, Malvern, UK). The samples were allowed to equilibrate for at least 15 min at each condition. At least three replicates were assessed for each sample to give an average hydrodynamic diameter and size distribution. The particle size distribution was referred to as the Span value and was calculated as follows [31,32,33]:

$$Span=\frac{D_{\text{V},90\%}-D_{\text{V},10\%}}{D_{\text{V},50\%}},$$

where *D*_v,90%_, *D*_v,50%_ and *D*_v,10%_ are the volume size diameters at 90%, 50% and 10% cumulative volumes, respectively. The smaller the span value indicates the narrower the size distribution.

The chemical structure was analyzed by Fourier transform infrared spectrometer (FTIR, NicoletiS50, Thermo Fisher Scientific Inc., Waltham, MA, USA) using a KBr tablet containing the microsphere powders.

Thermal analysis was performed by differential scanning calorimetry (DSC, TGA/DSC1, Mettler-Toledo Inc., Columbus, OH, USA) in a nitrogen atmosphere. Samples of approximately 4 mg were placed in aluminum sample pans and sealed. The first run was heated from 20 to 200 °C to remove the thermal history, and the second run was heated from 20 to 250 °C at a heating rate of 10 °C/min. An empty aluminum pan of an approximately equal weight was used as a reference.

### 2.5. Measurement of Thermo- and pH-responsive Behaviors of Microspheres

Thermo- and pH-responsive behaviors of the prepared microspheres were investigated by measuring the transmittance values of the microsphere aqueous dispersions at 575 nm using an UV-vis spectrometer. Microsphere dispersions were treated in an ultrasound instrument for 15 min before the measurements. During the thermo-responsive behaviors measurement, the temperatures were varied from 25 to 50 °C using a circulating water pump, and the heat rate was approximately 0.1–0.25 °C/min. The LCST value was judged to be the initial break point of the curve of transmittance *versus* temperature. For pH-responsive behavior measurements, a 0.1 M HCl solution and a 0.1 M NaOH solution were used to adjust the pH values of the dispersions. Every temperature or pH point was maintained for 10 min before reading the transmittance, and the measurements were repeated three times.

### 2.6. Examination of Microsphere Stability

Confocal laser scanning microscopy (CLSM, TCS SP5, Leica Microsystems, Wetzlar, Germany) was used to observe the microspheres in PBS solution (10 mM, pH 7.0), and the samples were labeled by Rhodamine 123 (Rh 123). At predetermined times, a small amount of microspheres (8 mg/mL) was removed from the samples and washed by water to remove the free PAAc chains. The microspheres were then added into the Rhodamine 123 solution (5 μg/mL) and incubated at 4 °C for 24 h. The labeled microspheres were separated from the unbound dye by centrifugation and were observed using CLSM at an excitation wavelength of 488 nm. For accuracy in the experiments, the same amount of microspheres, dye, and fluorescence intensity were maintained for the different samples.

## 3. Results and Discussion

### 3.1. Optimization of the Process Parameters for Preparing Microspheres with a Narrow Size Distribution

#### 3.1.1. Trans-Membrane Pressure

**Figure 2:**
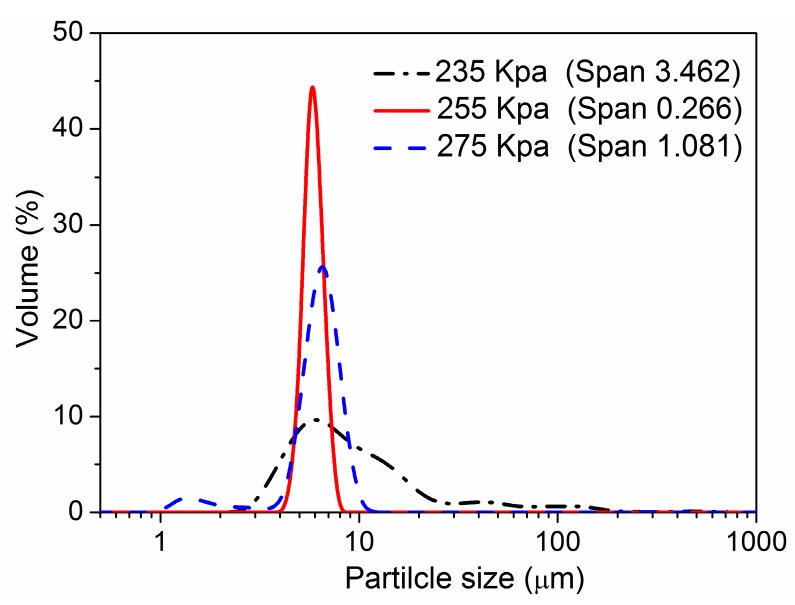
Size distributions of the semi-IPN3 microspheres prepared under different trans-membrane pressures.

In the premix membrane emulsification process, the preparation of uniform emulsion droplets is based on extruding the primary coarse emulsions through a membrane by a suitable pressure. Thus, the trans-membrane pressure is one of the crucial parameters influencing the size distribution. To investigate the effect of the pressure on particle size, the primary coarse emulsions of semi-IPN3 were pressed through a micro-porous membrane under different trans-membrane pressures, *i.e.*, 235, 255, and 275 kPa. The number of trans-membrane passes used was three, and the other parameters were the same as mentioned in Section 2.3. The relationship between the trans-membrane pressure and the size distribution of the microspheres is shown in Figure 2. It can been observed that the Span values of the resultant microspheres (3.462, 0.266, and 1.081 for 235, 255 and 275 kPa, respectively) were significantly influenced by pressure, and the narrowest size distribution of the microspheres was obtained at 255 kPa. Because they were at a lower pressure, the droplets of coarse emulsions were difficult to break and would pass through the membrane pores by changing their shape. In contrast, a higher pressure caused a faster pass-through rate and promoted the coarse emulsions to cross the membrane at a high speed. In this situation, a large amount of smaller droplets was obtained. Therefore, too low or too high of a trans-membrane pressure could lead to a broad size distribution of the microspheres, and 255 kPa was chosen as the suitable transmembrane pressure to prepare uniform semi-IPN microspheres in the following experiments.

#### 3.1.2. Number of Trans-Membrane Passes

**Figure 3:**
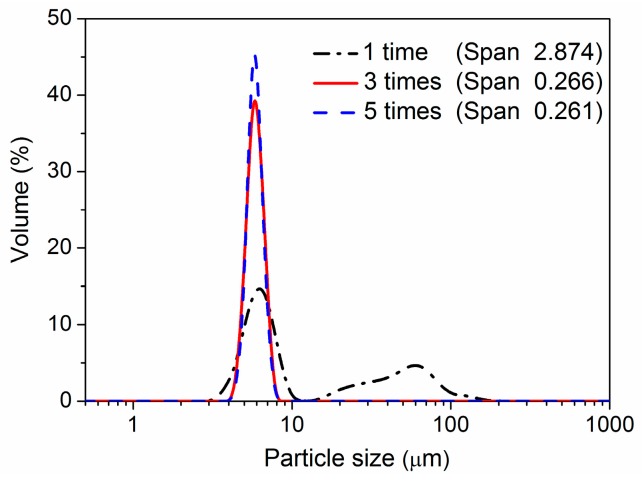
Size distributions of the semi-IPN3 microspheres prepared under different number of trans-membrane passes.

The number of trans-membrane passes is another key factor influencing the uniformity of microspheres. The primary coarse emulsions of semi-IPN3 were pressed through the micro-porous membrane for a different number of trans-membrane passes, and the other conditions were the same as mentioned in Section 2.3. As shown in Figure 3, a large amount of larger particles was obtained when the coarse emulsion was passed through the membrane only one time. The reason for this was that the droplets could not be fully emulsified by passing through the membrane pores only once. When the coarse emulsion was pressed through the membrane three and five times, the larger droplets were broken into smaller ones, and a narrower size distribution was obtained. The size distributions of the microspheres prepared by three and five trans-membrane passes were almost the same. For simplicity of operation, three passes were employed in the following experiments. Similar results have been reported in other studies [28,34].

### 3.2. Chemical Structure of the PNIPAM-PAAc Semi-IPN Microspheres

**Figure 4:**
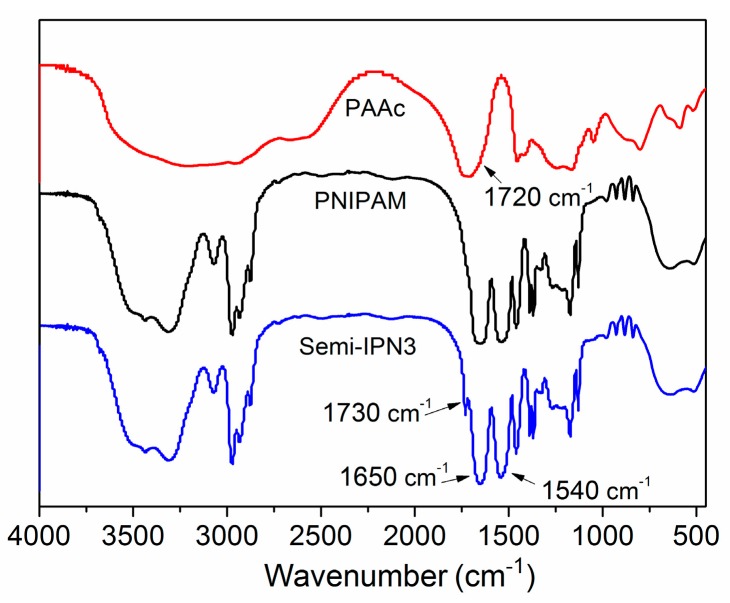
FT-IR spectra for the PAAc, PNIPAM, and semi-IPN3 microspheres.

FTIR spectroscopy was carried out to analyze the chemical compositions and molecular structures of the PNIPAM-PAAc semi-IPN microspheres. As depicted in Figure 4, the typical double peaks at 1650 and 1540 cm^−1^ could be assigned to the amide I band (C=O stretching) and the amide II band (N–H in-plane bending vibration) of PNIPAM, respectively. A band at 1720 cm^−1^ attributed to the stretching vibration of the carboxyl group (–COOH) could be found in the spectrum of PAAc. The spectra of semi-IPN3 showed the characteristic amide groups at 1650 and 1540 cm^−1^ of PNIPAM, and the carbonyl stretching bonds shifted to 1730 cm^−1^ of PAAc. A shift of wavenumbers to higher values on the carboxyl group in the semi-IPN microspheres was consistent with previously published reports [35,36]. These results were the consequence of the hydrogen bond between the carboxyl group of the PAAc chains and the amide group of the PNIPAM network, indicating that PNIPAM–PAAc semi-IPN microspheres were prepared successfully.

### 3.3. Particle Morphology and Size of the PNIPAM-PAAc Semi-IPN Microspheres

**Figure 5:**
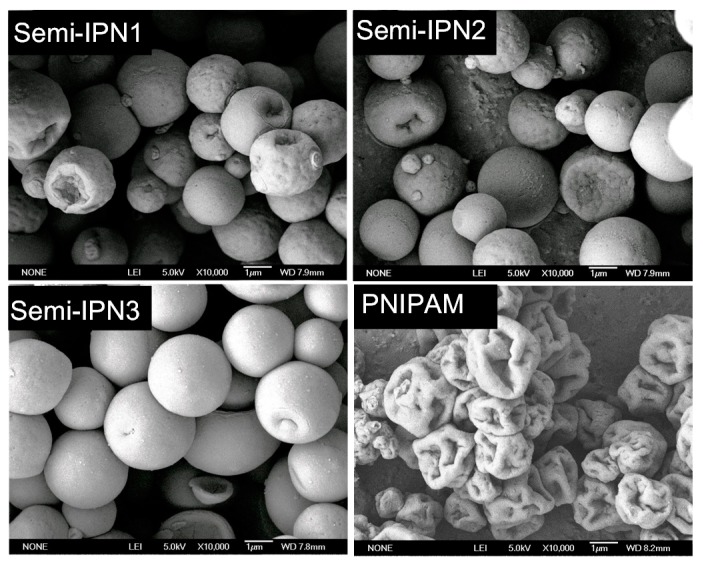
SEM images of semi-IPN and PNIPAM microspheres.

To observe the morphology of the microspheres in solid state, SEM measurements were performed. As shown in Figure 5, the surface of the PNIPAM microspheres were largely deformed compared with those of the PNIPAM–PAAc semi-IPN microspheres, which was due to the uneven collapse of the structure as a result of drying. These figures clearly show that the semi-IPN microspheres were more rigid than the PNIPAM microspheres. Some researchers have reported that the introduction of the semi-IPN structure and linear polymer chains could reinforce the unvarnished hydrogel network [37,38]. Thus, in the present system, it could be considered that the presence of the PAAc chains supported the PNIPAM network, resulting in less shrinkage during the dehydration process. Moreover, the surface of the semi-IPN3 microspheres was smoother than that of semi-IPN1 and semi-IPN2. The reason for this was that the increasing concentration of initiator led to a higher crosslinking density, increasing the rigidity of the microspheres [39].

**Figure 6:**
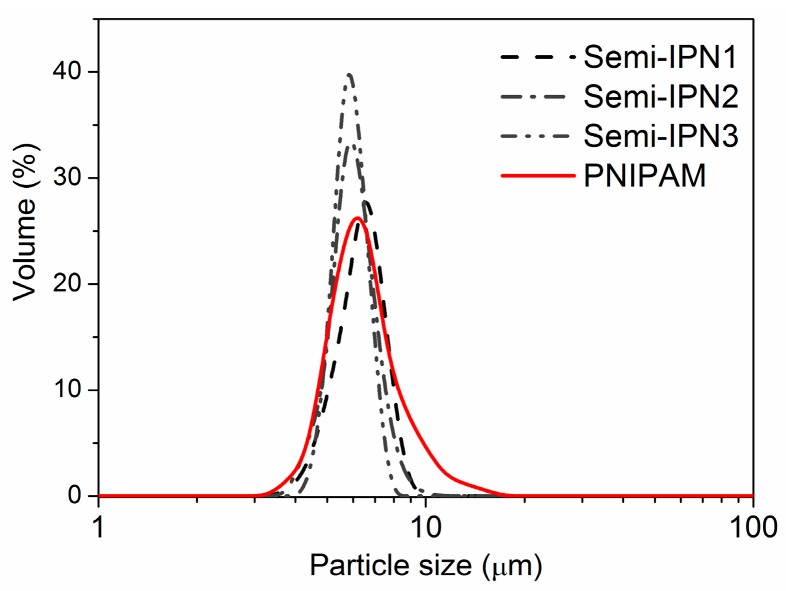
Particle size distribution of the semi-IPN and PNIPAM microspheres (10 mM, pH 7.0 PBS, 25 °C).

Figure 6 shows the particle size distributions of different microspheres, and Table 2 displays the particle sizes and the yield of the microspheres. It can be observed that the particle sizes of the semi-IPN microspheres (6.7, 6.3 and 6.2 μm, respectively) were similar to those of the PNIPAM microspheres (6.5 μm). However, the Span values of all the semi-IPN microspheres were smaller than those of the PNIPAM microspheres (1.571 ± 0.500), which meant that the size distribution of the semi-IPN microspheres was narrower than that of the PNIPAM microspheres. It has been reported that Span values of less than 1 were considered monosized distributions [40,41]. Thus, the uniform semi-IPN microspheres were obtained, and the average yield of the semi-IPN microspheres was 73%. Remarkably, the particle size distribution of semi-IPN1 was slightly different from those of semi-IPN2 and semi-IPN3, possibly because when the initiator concentration was 1.1 × 10^−2^ mol/L, the cross-linking density was smaller and the rigidity of the semi-IPN1 microspheres was lower. Hence, a tiny proportion of the microspheres could have been broken during the wash process, which increased the Span value.

### 3.4. TEM Observation of PNIPAM-PAAc Semi-IPN Microspheres

**Figure 7:**
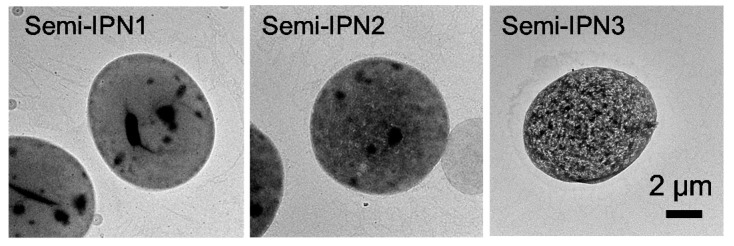
TEM images of semi-IPN with different initiator amounts (samples were labeled with uranyl acetate).

Transmission electron microscopy (TEM) was used for a further in-depth investigation of the microstructure of the PNIPAM–PAAc semi-IPN microspheres. Uranyl acetate was chosen to stain the microspheres before the measurement. Because PNIPAM cannot be stained, the black spots in the sample indicate the location of the PAAc domains. As shown in Figure 7, the amount of large-size black spots in the microspheres decreased from semi-IPN1 to semi-IPN3, and the amount of small-size black spots increased; moreover, a homogeneous texture associated with a network-like feature appeared in the semi-IPN3. These TEM pictures revealed that the increased initiator amount resulted in smaller PAAc and PNIPAM domains.

### 3.5. DSC Thermograms of the PNIPAM–PAAc Semi-IPN Microspheres

**Figure 8:**
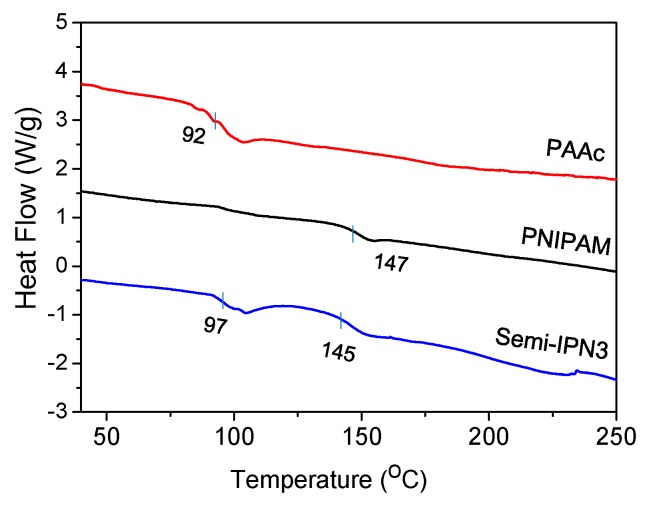
DSC curves of the PAAc, PNIPAM, and semi-IPN3 microspheres.

The glass transition temperature (*T*_g_) is a very important characteristic parameter of a polymer as it determines the miscibility of the polymer component. A DSC study was performed on the dried microspheres to obtain the *T*_g_ during the heating process, and the calorimetric endotherms are displayed in Figure 8. The *T*_g_ of PNIPAM and PAAc were 147 and 92 °C, respectively. PNIPAM–PAAc semi-IPN3 exhibited two *T*_g_ values of 97 and 145 °C, which was indicative of a phase-separated structure. Compared with pristine PNIPAM and PAAc microspheres, the *T*_g_ of PAAc and PNIPAM in the semi-IPN3 microspheres shifted towards each other. It has been reported that most IPNs form immiscible compositions during synthesis [42]. In addition, it is known from the literature that the *T*_g_ of the individual components shift towards each other, indicating a partial mixing of the networks [43,44]. Therefore, the DSC results of the present work showed that an incomplete compatible state existed between the two polymer networks. Meanwhile, the PAAc chains were entangled with the PNIPAM networks and were relatively independent of the PNIPAM networks.

### 3.6. Thermo- and pH-Responsive Behaviors of Microspheres

**Figure 9:**
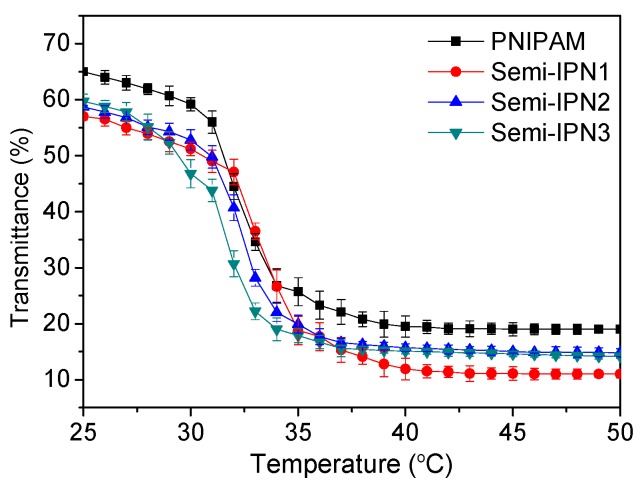
Transmittance of the PNIPAM and semi-IPN microsphere suspensions in different temperatures at pH 7.0.

The cloud-point method was used to observe the real-time change in the transmittance of microsphere suspensions in different temperature or pH conditions. The onset temperatures determined by transmittance *versus* the temperature curves, which corresponded to the first sign of microsphere aggregation in the solution, could be equal to the LCST [45]. Figure 9 shows the transmittance changes in the microsphere suspensions at temperatures ranging from 25 to 50 °C. For all semi-IPN samples, the transmittance values decreased significantly at temperatures approximately 32 °C, which was similar to the observations of the PNIPAM microspheres. Other researchers have also measured the transmittances of the suspensions of PNIPAM-*co*-AAc copolymer microspheres at various temperatures, and the results demonstrated that the AAc component could significantly increase the LCST value of the matrix [46]. However, in our experiment, an increase in the LCST values for the semi-IPNs was not observed. This result suggested that due to the chemical independence, the PAAc chains barely hindered the phase transition behavior of the PNIPAM network.

**Figure 10:**
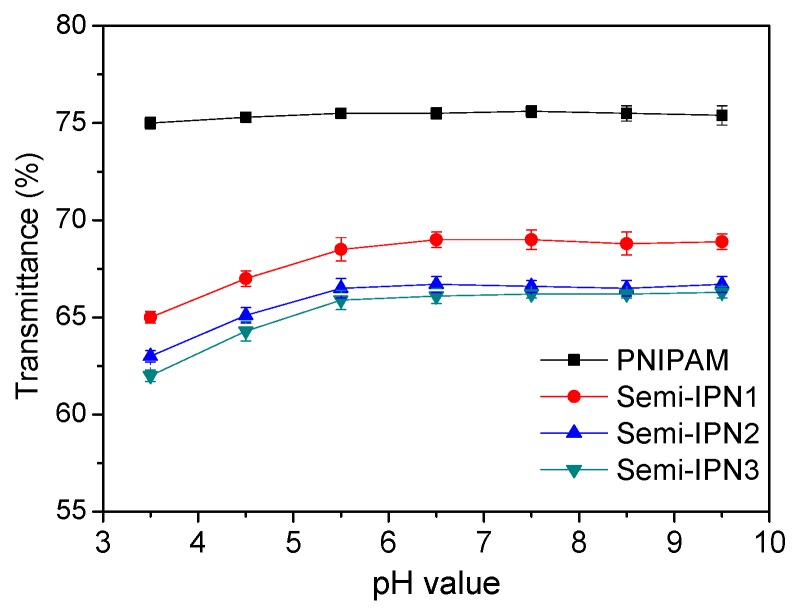
Transmittance of the PNIPAM and semi-IPN microsphere suspensions in different pH conditions at 25 °C.

To investigate the effect of pH on the swelling capacity of PNIPAM and semi-IPN microspheres, the microspheres were immersed in a buffer solution with a pH adjusted from 3.5 to 9.5. The transmittances of the microsphere suspensions as a function of pH are shown in Figure 10 and Table S1 (Supplementary Materials). No transmittance variation in the PNIPAM microsphere suspension was observed in the measured range, whereas the semi-IPN ones showed different transmittance values depending on the pH of the medium. When considered in detail, the curves in Figure 10 could be divided into two sections by pH 5.5. As the pH increased from 3.5 to 5.5, the transmittance increased obviously, indicating an increase in the particle diameter. Indeed, the particle size of microspheres at the various pH values was also measured, which showed similar results (Table S2 in Supplementary Materials). The reasons for this are as follows. When the pH was lower than the pKa of the PAAc component, due to the hydrogen bonding interactions between the amide group of PNIPAM and the carboxyl group of PAAc, the semi-IPN microspheres were in a shrunken state. As the pH increases above the pKa, the carboxyl groups were gradually dissociated, leading to a swelling of the microspheres derived from the increased osmotic pressure and electrostatic repulsion between the charged groups [47]. The transmittance increased with the pH until a plateau region was reached around pH 5.5, with similar behavior reported for PNIPAM–PAAc semi-IPN nanogels with inorganic clay as a crosslinker [19]. These results could be attributed to the completed disassociation of the carboxyl group at a pH higher than the pKa [48].The thermo-sensitive behaviors of the pristine PNIPAM and semi-IPN microspheres were also investigated at different pH values (Figure S1 in Supplementary Materials). It could be observed that as the pH increased from 4.5 to 8.5, the LCST values of the semi-IPN microspheres were increased slightly, whereas those of the PNIPAM microspheres were constant (Table S3 in Supplementary Materials). The results showed that the thermo-sensitive behaviors of the semi-IPN microspheres were barely influenced by the pH value and that the temperature-responsive and pH-responsive behaviors of the semi-IPN microspheres had little effect on each other.

### 3.7. Storage Stability of Microspheres

**Figure 11:**
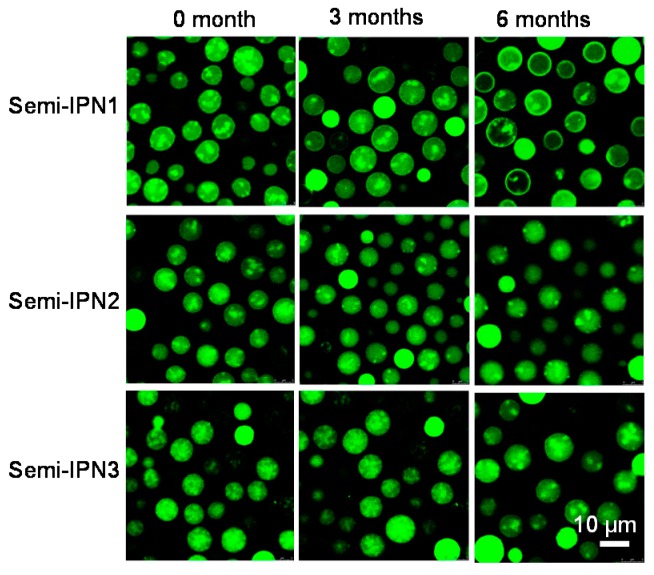
CLSM images of the semi-IPN microspheres at different storage times (4 °C, 10 mM pH 7.0 PBS solution; the samples were labeled with Rh 123).

Unlike covalently cross-linked structures, physical entanglement is usually not permanent [49]. In the semi-IPNs system, the PAAc chains and PNIPAM network interacted with each other by hydrogen bond and physical entanglement; thus, it was possible for a fraction of the PAAc chains to diffuse out of semi-IPN microspheres during the storage period. In the enzyme immobilization process, the medium is always a pH 7.0 PBS solution, and the microspheres are usually kept in this solution. The change of functional groups in the microspheres greatly influenced the morphology and the dispersibility of the microspheres, and thus would change the configuration of the enzyme molecules. Taking this into account, an investigation of the morphology and particle size was used to study the stability and dispersibility of microspheres. As shown in Figure 11, all the semi-IPN microspheres were labeled well as Rhodamine 123 (Rh123) has amino groups that would interact with the carboxyl groups of the microspheres. After three months, no obvious change in the fluorescence distribution of the samples was observed. Because only PAAc chains were labeled by Rh123 in the microspheres, the leaching phenomenon of PAAc was hardly observed, which implied that the PAAc chains stably existed in the semi-IPN microspheres over the three months. With a storage time of up to six months, all the samples still had good dispersibility. The morphologies of the semi-IPN2 and semi-IPN3 microspheres were almost the same as the original, whereas there was a little change in the fluorescence distribution in the semi-IPN1. The fluorescence tended to distribute near the surface of the semi-IPN1 microspheres, suggesting that the PAAc chains in the semi-IPN1 could diffuse out of the microspheres easier than the other samples. Combined with the TEM images, the reason for this might be a loose structure in the semi-IPN1.

**Figure 12:**
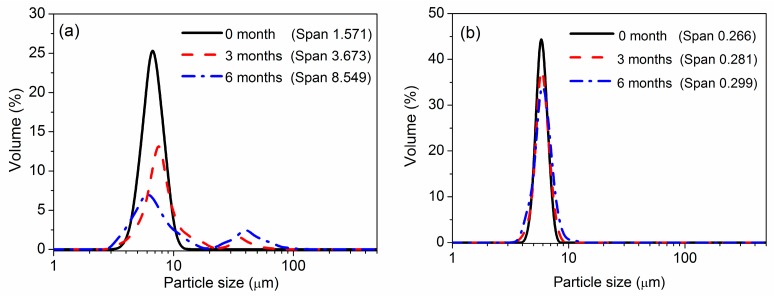
Size distribution of the PNIPAM (**a**) and semi-IPN3 (**b**) microspheres at different storage times (4 °C, 10 mM pH 7.0 PBS solution).

Figure 12 presents the particle size distribution of the PNIPAM and semi-IPN microspheres at different storage times. It was observed that both the PNIPAM and the semi-IPN microspheres had a narrow size distribution at the initial time. After three months, a bigger size distribution of the PNIPAM microspheres started to emerge. Six months later, the original size distribution of the PNIPAM microspheres disappeared and a bimodal size distribution appeared. The bigger size distribution indicated that the PNIPAM appeared to form aggregates over a prolonged time. The generation of PNIPAM microsphere aggregation might be derived from the absence of charged groups. In contrast, the semi-IPN microspheres retained a narrow size distribution during the whole measurement period. These results, combined with the CLSM images, indicated that the motility of the PAAc chains were restricted by the PNIPAM network in the semi-IPN microspheres, and the PAAc chains in turn improved the dispersibility of the microspheres. In general, the particle size and morphology of the semi-IPN microspheres were relatively stable compared with the PNIPAM microspheres over several months, which indicated that the storage stabilities of the semi-IPN microspheres were more desirable than the PNIPAM microspheres.

## 4. Conclusions

In this work, we developed a novel one-step method to synthesize uniform dual stimuli-responsive microspheres with semi-IPN structures. By polymerization of NIPAM monomers with an MBA cross-linkers in an AAc aqueous solution, PNIPAM–PAAc semi-IPN microspheres were formed directly. The morphologies of the dried microspheres were observed by SEM. The results revealed that the introduction of the semi-IPN structure and the PAAc chains could reinforce the rigidity of the hydrogel network, and uniform and spherical semi-IPN microspheres were obtained. Both the PNIPAM and semi-IPN microspheres possessed a particle size of approximately 6 μm, and the particle size distribution of the latter was narrower than the former. The semi-IPN microspheres underwent a similar sharp volume phase transition at 32 °C, which was barely influenced by the interpenetration of hydrophilic PAAc. The particle sizes of the semi-IPN microspheres changed with increasing pH values, which was due to the contribution of the PAAc. In addition, the temperature-responsive and pH-responsive properties had little interference with each other. After storage in a PBS solution at 4 °C for 6 months, the morphology and particle size of the semi-IPN microspheres were more stable than those of the PNIPAM microspheres. These studies on network formation and properties provide a reference for designing dual-responsive micro-size hydrogel particles and microspheres with a uniform size, which have various advantages for use in different applications, such as use as drug carriers and enzyme support.

## Figures and Tables

**Table 1:** Recipes for the preparation of PNIPAM-based microspheres.

Sample Code	NIPAM (×10^−1^ mol/L)	PAAc (×10^−5^ mol/L)	MBA (×10^−2^ mol/L)	APS (×10^−2^ mol/L)	TEMED (×10^−3^ mol)
PNIPAM	7.1	0	3.2	1.1	6.7
semi-IPN1	7.1	3.4	3.2	1.1	6.7
semi-IPN2	7.1	3.4	3.2	2.2	13.4
semi-IPN3	7.1	3.4	3.2	3.3	20.1

**Table 2:** Particle sizes and Span values of the different microspheres (10 mM, pH 7.0 PBS, 25 °C).

Sample	Particle Size (μm)	Span
PNIPAM	6.5 ± 1.7	1.571 ± 0.500
Semi-IPN1	6.7 ± 2.5	0.649 ± 0.219
Semi-IPN2	6.3 ± 1.1	0.272 ± 0.132
Semi-IPN3	6.2 ± 0.7	0.266 ± 0.112

## Supplementary Materials

Supplementary Materials can be found at www.mdpi.com/2073-4360/8/3/90/s1.

## References

[1] Gil E., Hudson S. (2004). Stimuli-reponsive polymers and their bioconjugates. Prog. Polym. Sci..

[2] Ono Y., Shikata T. (2006). Hydration and dynamic behavior of poly(*N*-isopropylacrylamide)s in aqueous solution. J. Am. Chem. Soc..

[3] Pinheiro J.P., Moura L., Fokkink R., Farinha J.P. (2012). Preparation and characterization of low dispersity anionic multiresponsive core-shell polymer nanoparticles. Langmuir.

[4] Hendrickson G.R., Smith M.H., South A.B., Lyon L.A. (2010). Design of multiresponsive hydrogel particles and assemblies. Adv. Funct. Mater..

[5] Thorne J.B., Vine G.J., Snowden M.J. (2011). Microgel applications and commercial considerations. Colloid. Polym. Sci..

[6] Welsch N., Becker A.L., Dzubiella J., Ballauff M. (2012). Core–shell microgels as “smart” carriers for enzymes. Soft Matter.

[7] Wu Q., Su T., Mao Y., Wang Q. (2013). Thermal responsive microgels as recyclable carriers to immobilize active proteins with enhanced nonaqueous biocatalytic performance. Chem. Commun..

[8] Kawaguchi H. (2014). Thermoresponsive microhydrogels: Preparation, properties and applications. Polym. Int..

[9] Kratz K., Hellweg T., Eimer W. (2000). Influence of charge density on the swelling of colloidal poly(*N*-isopropylacrylamide-*co*-acrylic acid) microgels. Colloids Surf. A.

[10] Burmistrova A., Richter M., Eisele M., Üzüm C., von Klitzing R. (2011). The effect of co-monomer content on the swelling/shrinking and mechanical behaviour of individually adsorbed PNIPAM microgel particles. Polymers.

[11] Si T., Wang Y., Wei W., Lv P., Ma G., Su Z. (2011). Effect of acrylic acid weight percentage on the pore size in poly(*N*-isopropylacrylamide-*co*-acrylic acid) microspheres. React. Funct. Polym..

[12] Li Z., Shen J., Ma H., Lu X., Shi M., Li N., Ye M. (2012). Preparation and characterization of sodium alginate/poly(*N*-isopropylacrylamide)/clay semi-IPN magnetic hydrogels. Polym. Bull..

[13] Hoare T., Pelton R. (2004). Highly pH and temperature responsive microgels functionalized with vinylacetic acid. Macromolecules.

[14] Koul V., Mohamed R., Kuckling D., Adler H.J., Choudhary V. (2011). Interpenetrating polymer network (IPN) nanogels based on gelatin and poly(acrylic acid) by inverse miniemulsion technique: Synthesis and characterization. Colloids Surf. B.

[15] Berger J., Reist M., Mayer J.M., Felt O., Peppas N.A., Gurny R. (2004). Structure and interactions in covalently and ionically crosslinked chitosan hydrogels for biomedical applications. Eur. J. Pharm. Biopharm..

[16] Lipatov Y.S., Alekseeva T.T. (2007). Phase-separated interpenetrating polymer networks. Adv. Polym. Sci..

[17] Xia X., Hu Z. (2004). Synthesis and light scattering study of microgels with interpenetrating polymer networks. Langmuir.

[18] Hu Z., Xia X. (2004). Hydrogel nanoparticle dispersions with inverse thermoreversible gelation. Adv. Mater..

[19] Ma J., Fan B., Liang B., Xu J. (2010). Synthesis and characterization of poly(*N*-isopropylacrylamide)/poly(acrylic acid) semi-IPN nanocomposite microgels. J. Colloid Interf. Sci..

[20] Xing Z., Wang C., Yan J., Zhang L., Li L., Zha L. (2010). pH/Temperature dual stimuli-responsive microcapsules with interpenetrating polymer network structure. Colloid. Polym. Sci..

[21] Xing Z., Wang C., Yan J., Zhang L., Li L., Zha L. (2011). Dual stimuli responsive hollow nanogels with IPN structure for temperature controlling drug loading and pH triggering drug release. Soft Matter.

[22] Ahmad H., Nurunnabi M., Rahman M.M., Kumar K., Tauer K., Minami H., Gafur M.A. (2014). Magnetically doped multi stimuli-responsive hydrogel microspheres with IPN structure and application in dye removal. Colloids Surf. A.

[23] Jones C.D., Lyon L.A. (2000). Synthesis and characterization of multiresponsive core-shell microgels. Macromolecules.

[24] Nilsson C., Birnbaum S., Nilsson S. (2011). Nanoparticle-based pseudostationary phases in CEC: A breakthrough in protein analysis?. Electrophoresis.

[25] Kwok M.-H., Li Z.-F., Ngai T. (2013). Controlling the synthesis and characterization of micrometer-sized PNIPAM microgels with tailored morphologies. Langmuir.

[26] Ma G.-H., Sone H., Omi S. (2004). Preparation of uniform-sized polystyrene–polyacrylamide composite microspheres from a wow emulsion by membrane emulsification. Macromolecules.

[27] Wei Y., Wang Y.-X., Wang W., Ho S.V., Wei W., Ma G.-H. (2011). mPEG–PLA microspheres with narrow size distribution increase the controlled release effect of recombinant human growth hormone. J. Mater. Chem..

[28] Wang Y.-X., Qin J., Wei Y., Li C.-P., Ma G.-H. (2013). Preparation strategies of thermo-sensitive P(NIPAM-*co*-AA) microspheres with narrow size distribution. Powder Technol..

[29] Ma G.-H. (2014). Microencapsulation of protein drugs for drug delivery: Strategy, preparation, and applications. J. Control Release.

[30] Myung D., Koh W., Ko J., Hu Y., Carrasco M., Noolandi J., Ta C.N., Frank C.W. (2007). Biomimetic strain hardening in interpenetrating polymer network hydrogels. Polymer.

[31] Qi F., Wu J., Fan Q.-Z., He F., Tian G.-F., Yang T.Y., Ma G.-H., Su Z.-G. (2013). Preparation of uniform-sized exenatide-loaded PLGA microspheres as long-effective release system with high encapsulation efficiency and bio-stability. Colloid. Surface B.

[32] Fuminori I., Kimiko M. (2004). Preparation and properties of monodispersed rifampicin-loaded poly(lactide-*co*-glycolide) microspheres. Colloids Surfaces B Biointerf..

[33] Arash K., Rassoul K. (2011). Effect of Alyssum homolocarpum seed gum, Tween 80 and NaCl on droplets characteristics, flow properties and physical stability of ultrasonically prepared corn oil-in-water emulsions. Food Hydrocolloids.

[34] Ma G.-H., Yang J., Lv P.-P., Wang L.-Y., Wei W., Tian R., Wu J., Su Z.-G. (2010). Preparation of uniform microspheres and microcapsules by modified emulsification process. Macromol. Symp..

[35] Xiao X., Zhuo R., Xu J., Chen L. (2006). Effects of reaction temperature and reaction time on positive thermosensitivity of microspheres with poly(acrylamide)/poly(acrylic acid) IPN shells. Eur. Polym. J..

[36] Burillo G., Briones M., Adem E. (2007). IPN’s of acrylic acid and *N*-isopropylacrylamide by gamma and electron beam irradiation. Nucl. Instrum. Methods Phys. Res. Sect. B.

[37] Djonlagi J., Petrovi Z.S. (2004). Semi-interpenetrating polymer networks composed of poly(*N*-isopropyl acrylamide) and polyacrylamide hydrogels. J. Polym. Sci. Part B.

[38] Zhao S.-P., Ma D., Zhang L.-M. (2006). New semi-interpenetrating network hydrogels: Synthesis, characterization and properties. Macromol. Biosci..

[39] Potorac S., Popa M., Verestiuc L., le Cerf D. (2012). New semi-IPN scaffolds based on HEMA and collagen modified with itaconic anhydride. Mater. Lett..

[40] Williams R.A., Peng S.J., Wheeler D.A., Morley N.C., Taylor D., Whalley M., Houldsworth D.W. (1998). Controlled production of emulsions using a crossflow membrane. Chem. Eng. Res. Des..

[41] Mariana P.-H., Richard G.-H. (2014). Membrane emulsification for the production of uniform poly-*N*-isopropylacrylamide-coated alginate particles using internal gelation. Chem. Eng. Res. Des..

[42] John J., Klepac D., Didović M., Sandesh C.J., Liu Y., Raju K.V.S.N., Pius A., Valić S., Thomas S. (2010). Main chain and segmental dynamics of semi interpenetrating polymer networks based on polyisoprene and poly(methyl methacrylate). Polymer.

[43] Liu T.-Y., Lin W.-C., Huang L.-Y., Chen S.-Y., Yang M.-C. (2005). Surface characteristics and hemocompatibility of PAN/PVDF blend membranes. Polym. Adv. Technol..

[44] Thimma Reddy T., Takahara A. (2009). Simultaneous and sequential micro-porous semi-interpenetrating polymer network hydrogel films for drug delivery and wound dressing applications. Polymer.

[45] Fundueanu G., Constantin M., Asmarandei I., Bucatariu S., Harabagiu V., Ascenzi P., Simionescu B.C. (2013). Poly(*N*-isopropylacrylamide-*co*-hydroxyethylacrylamide) thermosensitive microspheres: The size of microgels dictates the pulsatile release mechanism. Eur. J. Pharm. Biopharm..

[46] Khan A. (2007). Preparation and characterization of *N*-isopropylacrylamide/acrylic acid copolymer core-shell microgel particles. J. Colloid Interface Sci..

[47] Liu X.-Y., Guo H., Zha L.-S. (2012). Study of pH/temperature dual stimuli-responsive nanogels with interpenetrating polymer network structure. Polym. Int..

[48] Chen Y., Ding D., Mao Z., He Y., Hu Y., Wu W., Jiang X. (2008). Synthesis of hydroxypropylcellulose-poly(acrylic acid) particles with semi-interpenetrating polymer network structure. Biomacromolecules.

[49] Myung D., Waters D., Wiseman M., Duhamel P., Noolandi J., Ta C.N., Frank C.W. (2008). Progress in the development of interpenetrating polymer network hydrogels. Polym. Adv. Technol..

