# Supplementary material for: Preparation of Uniform-Sized and Dual Stimuli-Responsive Microspheres of Poly(N-Isopropylacrylamide)/Poly(Acrylic acid) with Semi-IPN Structure by One-Step Method
Source: Polymers (Basel). 2016 Mar 17;8(3):90. doi: 10.3390/polym8030090 (PMC6432549; doi:10.3390/polym8030090)
Supplement: Supplementary file 1 [file polymers-08-00090-s001.pdf]

Supplementary Materials: Preparation of Uniform-Sized and Dual Stimuli-Responsive Microspheres of Poly(*N*-Isopropylacrylamide)/ Poly(Acrylic Acid) with SemiIPN Structure by One-Step Method

En-Ping Lai, Yu-Xia Wang, Yi Wei and Guang Li

Table S1. Transmittance value of PNIPAM and semi-IPN microspheres at different pH values.

pH value	PNIPAM	Semi-IPN1	Semi-IPN2	Semi-IPN3
3.5	75	65	63	62
4.5	75.3	67	65.1	64.3
5.5	75.5	68.5	66.5	65.9
6.5	75.5	69	66.7	66.1
7.5	75.6	69	66.6	66.2
8.5	75.5	68.8	66.5	66.2
9.5	75.4	68.9	66.7	66.3

Table S2. Particle size of Semi-IPN microspheres at various pH values.

Samples	Particle size (μm)					
	pH 3.5	pH 4.5	pH 5.5	pH 6.5	pH 7.5	pH 8.5
Semi-IPN1	4.59 ± 1.02	5.73 ± 0.86	6.58 ± 0.98	6.62 ± 0.52	6.76 ± 1.53	6.81 ± 1.34
Semi-IPN2	3.13 ± 1.24	5.22 ± 0.91	5.93 ± 0.74	6.09 ± 1.77	6.3 ± 1.19	6.45 ± 1.65
Semi-IPN3	2.76 ± 0.33	4.52 ± 1.42	5.97 ± 0.48	6.12 ± 0.81	6.2 ± 0.73	6.17 ± 1.90

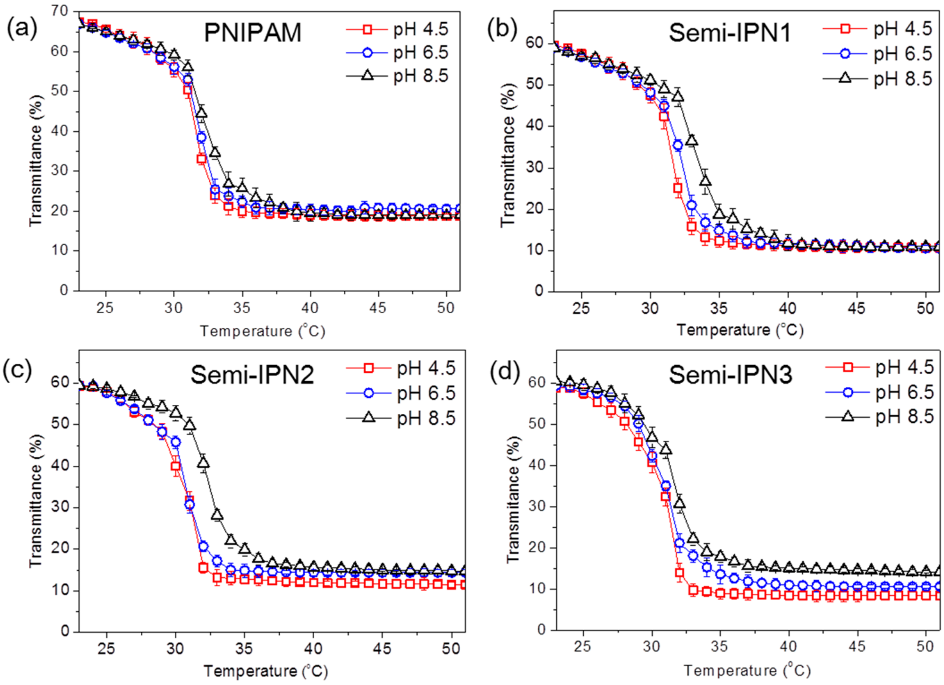

Figure S1. Transmittances of (**a**) PNIPAM, (**b**) semi-IPN1, (**c**) semi-IPN2 and (**d**) semi-IPN3 microsphere suspensions as a function of temperature at different pH values.

Table S3. LCST values of resultant microspheres at different pH values.

**Samples**	**LCST**		
	**pH 4.5**	**pH 6.5**	**pH 8.5**
PNIPAM	31.5	31.5	31.6
Semi-IPN1	31.7	31.9	32.3
Semi-IPN2	31.7	32.0	32.1
Semi-IPN3	31.8	32.2	32.5

© 2016 by the authors; licensee MDPI, Basel, Switzerland. This article is an open access article distributed under the terms and conditions of the Creative Commons by Attribution (CC-BY) license (http://creativecommons.org/licenses/by/4.0/).
